# Supplementary figures and images for: Global Chromosome Topology and the Two-Component Systems in Concerted Manner Regulate Transcription in Streptomyces
Source: mSystems. 2021 Nov 16;6(6):e01142-21. doi: 10.1128/mSystems.01142-21 (PMC8594442; doi:10.1128/mSystems.01142-21)

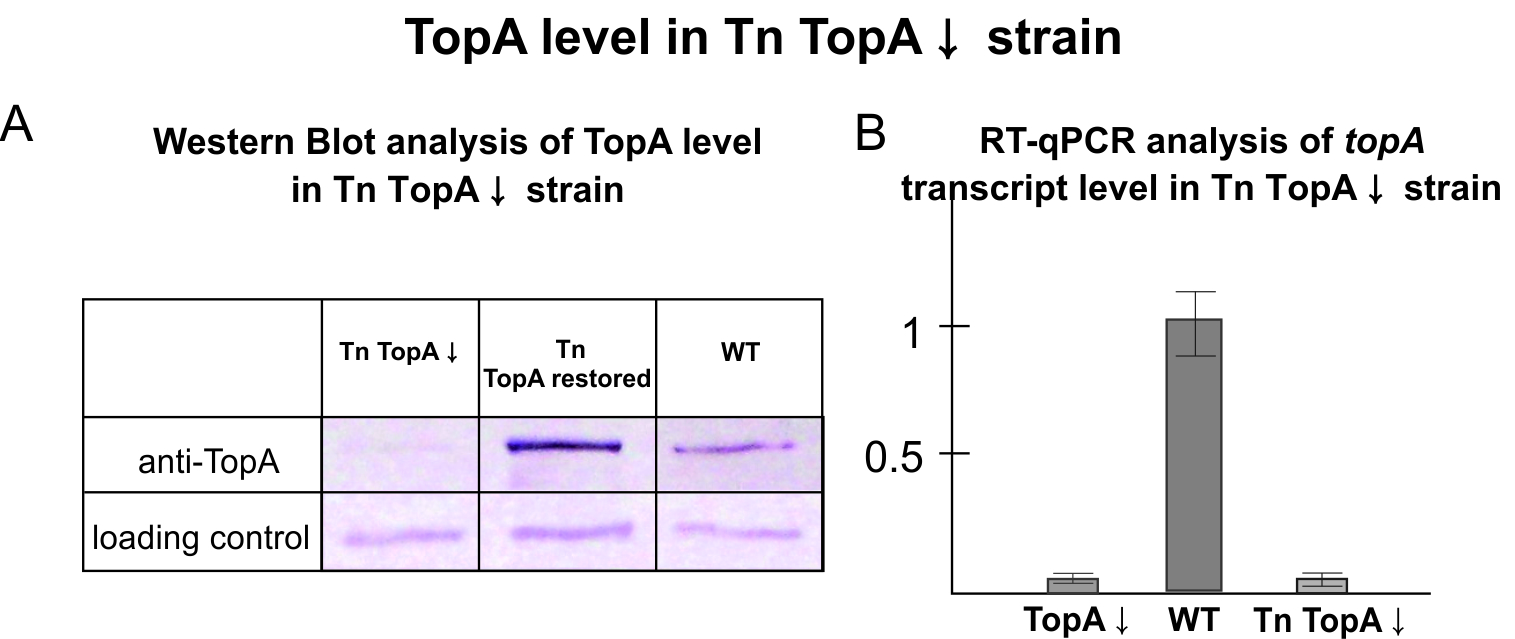

Supplement: FIG S1 [file msystems.01142-21-sf001.jpg]

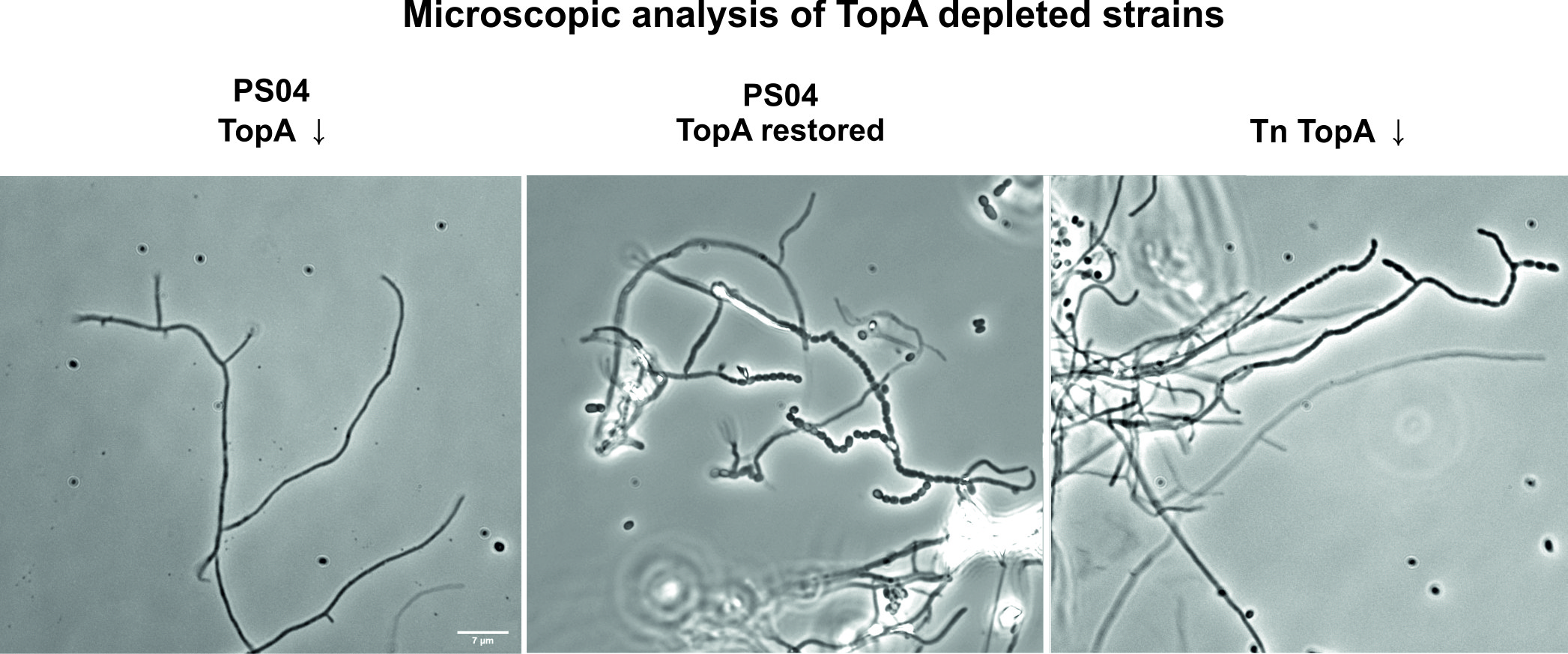

Supplement: FIG S2 [file msystems.01142-21-sf002.jpg]

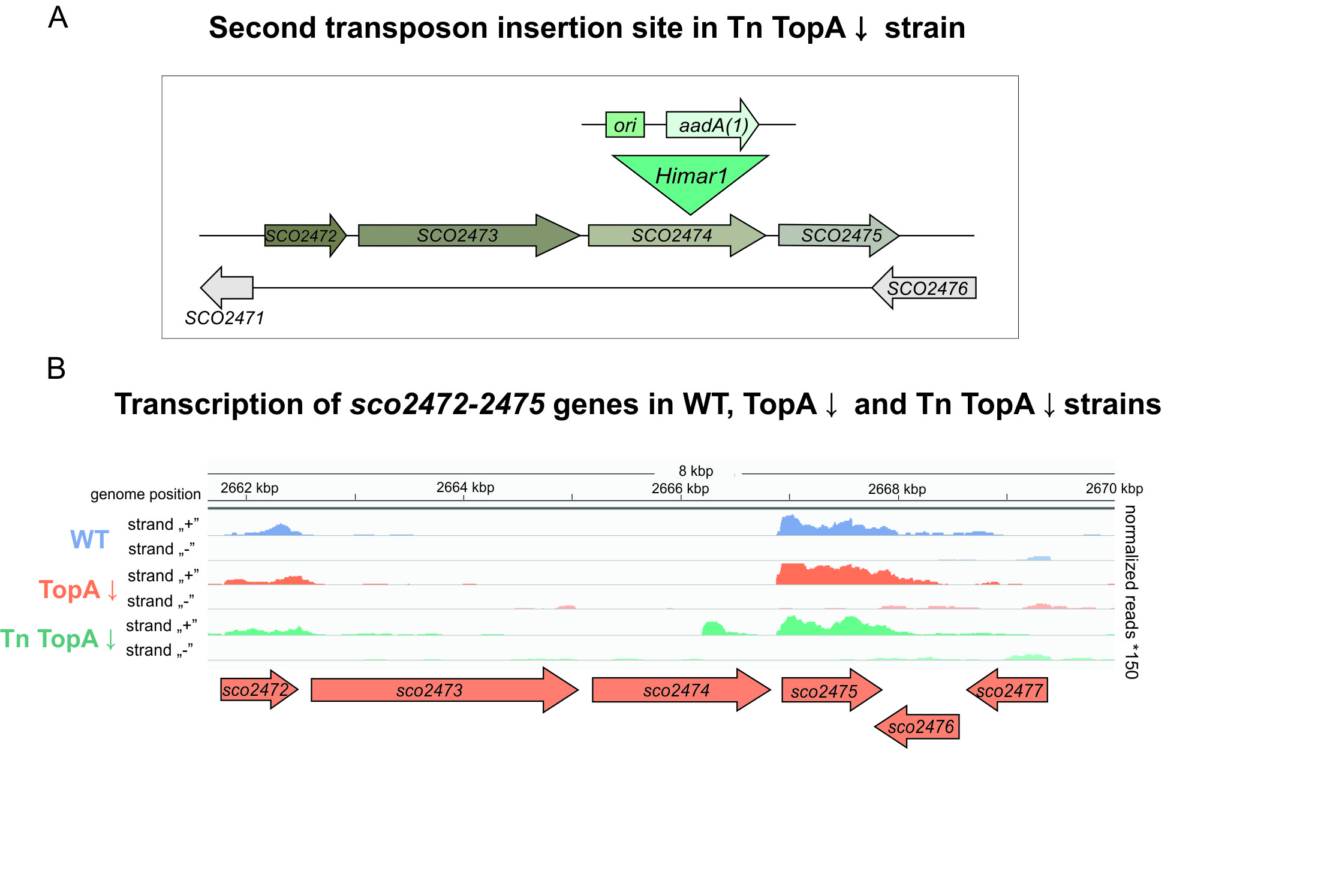

Supplement: FIG S3 [file msystems.01142-21-sf003.jpg]

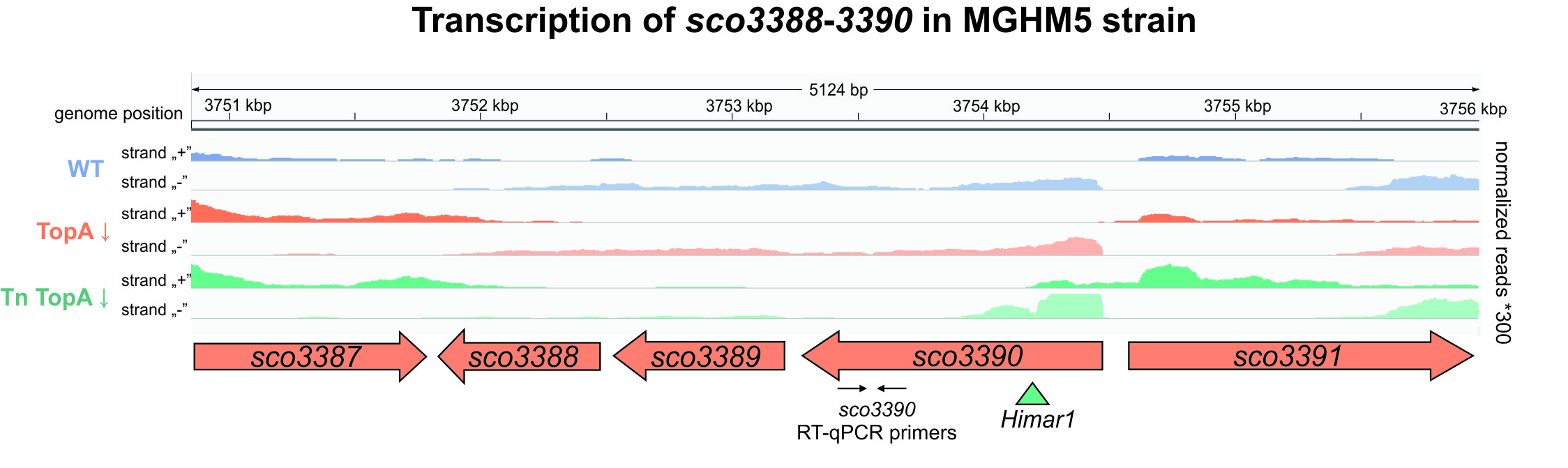

Supplement: FIG S4 [file msystems.01142-21-sf004.jpg]

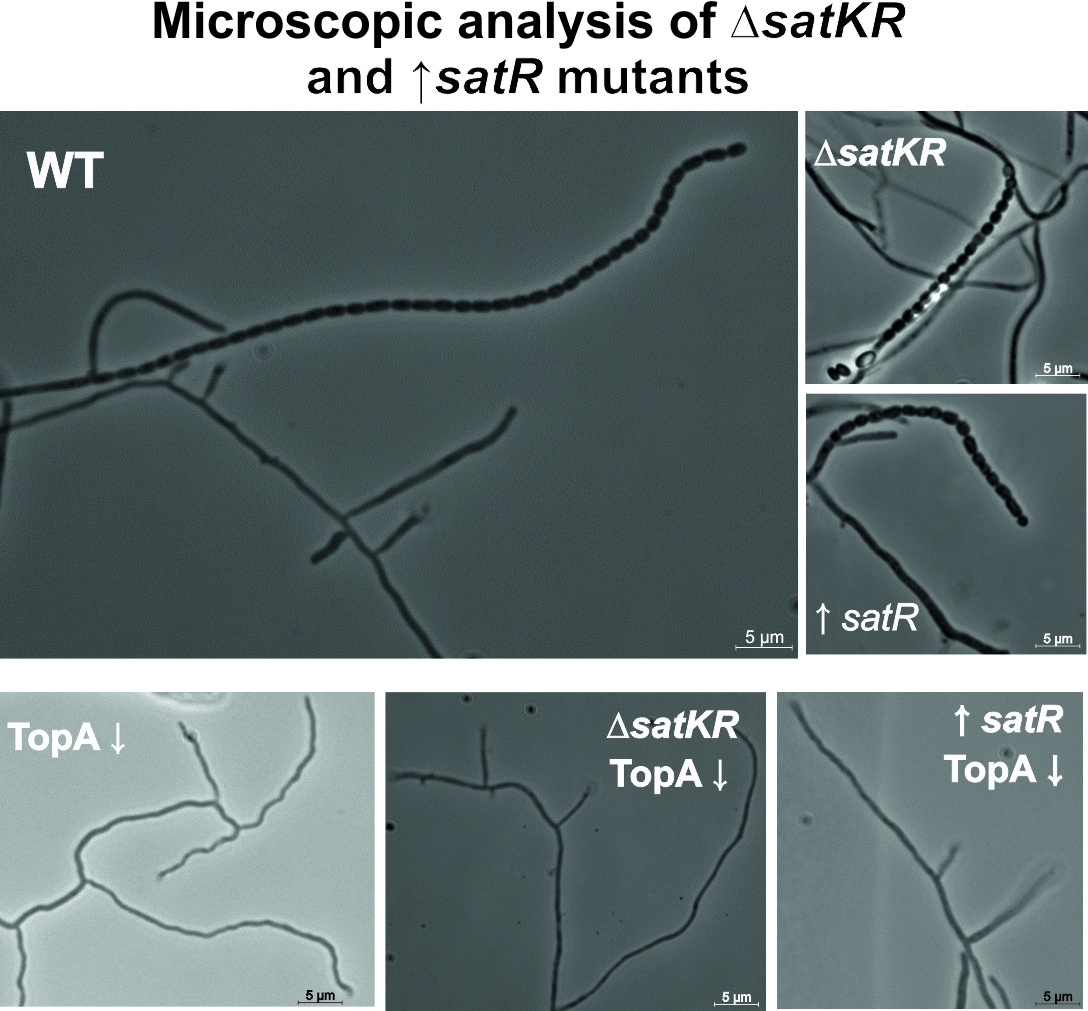

Supplement: FIG S5 [file msystems.01142-21-sf005.jpg]

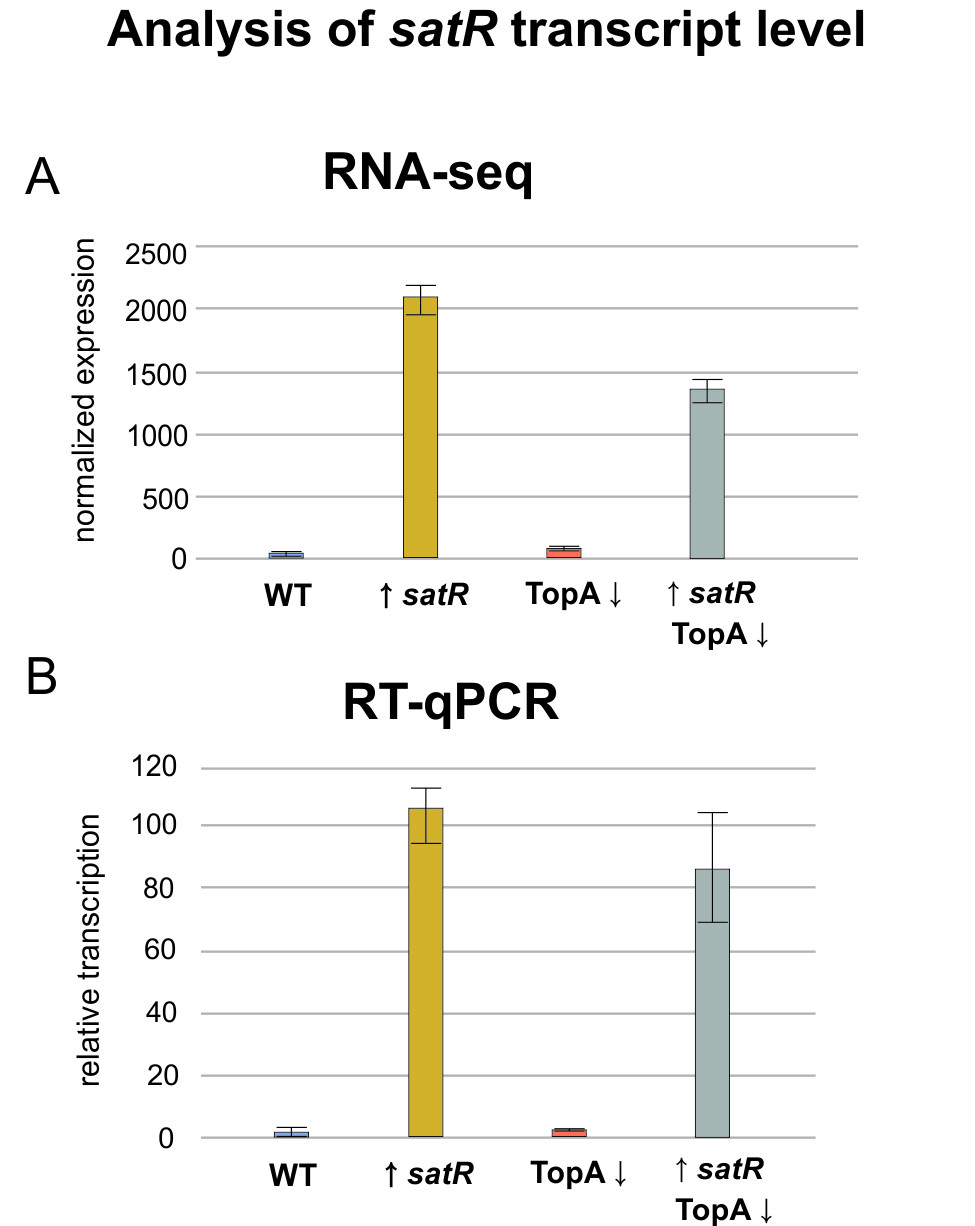

Supplement: FIG S6 [file msystems.01142-21-sf006.jpg]

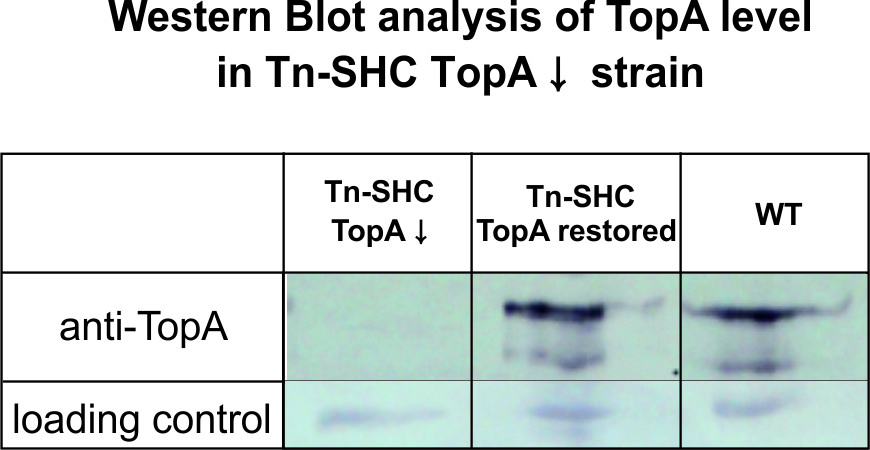

Supplement: FIG S7 [file msystems.01142-21-sf007.jpg]

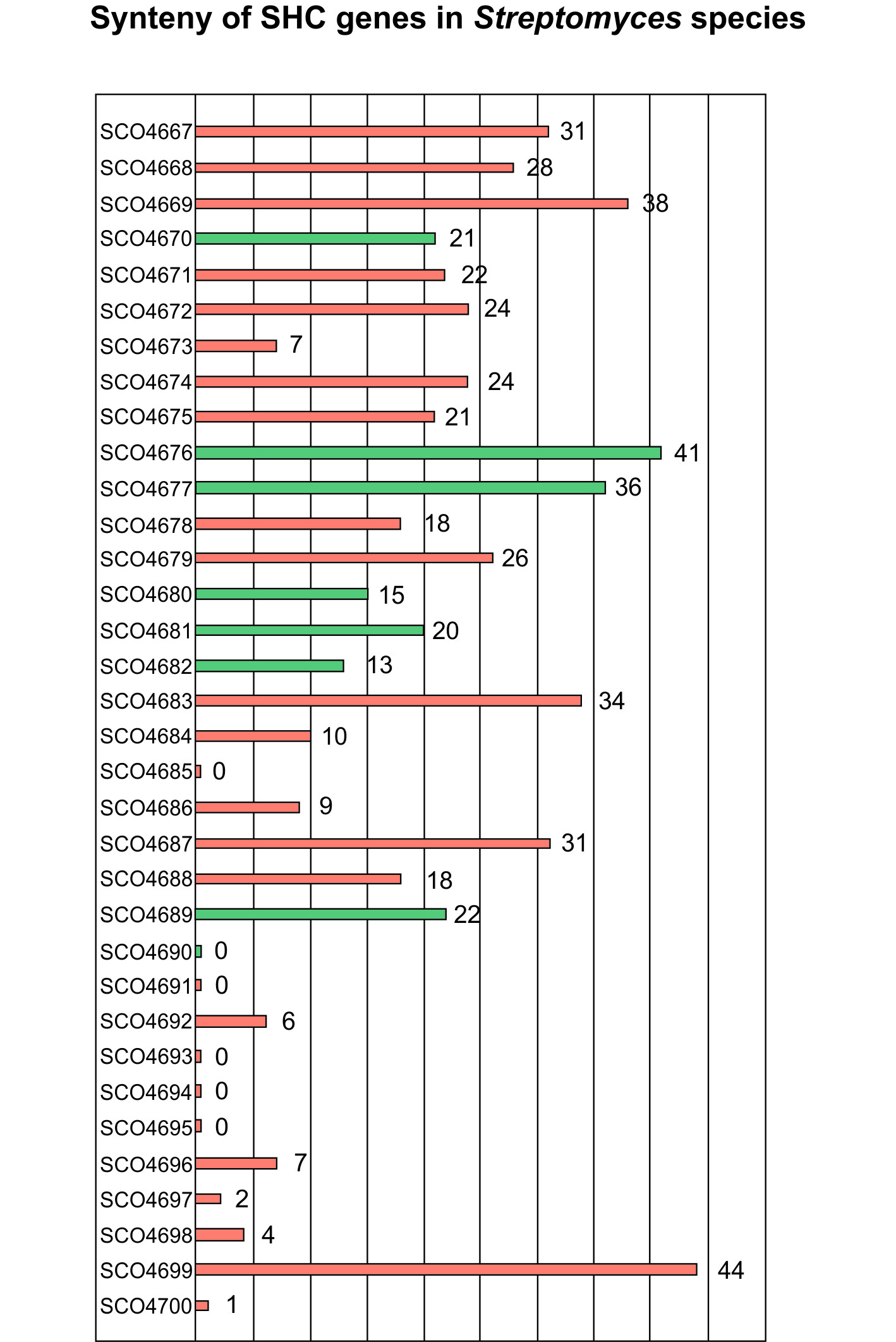

Supplement: FIG S8 [file msystems.01142-21-sf008.jpg]
