# Supplementary material for: Global Chromosome Topology and the Two-Component Systems in Concerted Manner Regulate Transcription in Streptomyces
Source: mSystems. 2021 Nov 16;6(6):e01142-21. doi: 10.1128/mSystems.01142-21 (PMC8594442; doi:10.1128/mSystems.01142-21)
Supplement: TEXT S1 [file msystems.01142-21-s0001.pdf]

Supplementary file 1

A. Strains used in the study

Name	Genotype	Source or reference
WT	*S. coelicolor* M145 SCP1$^-$ SCP2$^-$	(1)
PS04<br><br>(TopA↓, p_{tipA}*topA*)	M145 Δ*topA::scar* attBΦC31::pIJ6902*topA*	(2)
PS04-Tnlib	PS04 Himar1 mutant library	(this study)
MGHM5<br><br>(Tn TopA↓)	PS04 *sco3390*::*Himar1*, *sco2474*::*Himar1*	(this study)
MGHM14<br><br>(Tn-SHC TopA↓)	PS04 *sco4699*::*Himar1*	(this study)
MS10	M145 pWHM3Hyg	(3)
MS11	PS04 pWHM3Hyg	(3)
MGHM5_RP	MGHM5 pWHM3Hyg	(this study)
MGM10	M145 Δ*sco3390*	(this study)
MGP10	PS04 Δ*sco3390*	(this study)
MGM12	M145 Δ*sco3390-3389*	(this study)
MGP12	PS04 Δ*sco3390-3389*	(this study)
MGM11	M145 attBΦBT1::pIJ10257*sco3389*	(this study)
MGP11	PS04 attBΦBT1::pIJ10257*sco3389*	(this study)

MGP14	MGP10 attBΦBT1::pIJ10257*sco3389*	(this study)
MB01	M145 Δ*sco4667-4668*::*hyg*	(this study)
MB02	PS04 Δ*sco4667-4668::hyg*	(this study)
MGP20	*sco4699*::*hyg*	(this study)

B. Strains construction

Deletions of *sco3390-89* genes (strains MGM12 and MGP12) were performed using CRISPR-Cas9 method and procedures described by (4). Annealed oligo3390_BbsI_fw and oligo3390_BbsI_rv oligonucleotides were cloned into pCRISPomyces-2 vector as the template for sgRNA (yielding pCRISPomyces-2_oligo3390). Next, the template sequence for homologous recombination-driven repair was amplified on the *S. coelicolor* chromosome using oligonucleotides XbaI_fw_sco3390_frI and del_rv_sco3390_frI for the fragment upstream the deleted genes; and del_fw_sco3390_frII and XbaI_rv_sco3390_frII for the fragment downstream. The obtained fragments (length 1145 bp and 1117 bp, respectively) were inserted in pCRISPomyces-2_oligo3390 in XbaI site using SLIC method, yielding pCRISPomyces-2_Δ*sco3390-3389* plasmid. PCR targeting method (5)was then used to exchange apramycin resistance gene in pCRISPomyces-2_Δ*sco3390-3389* plasmid for hygromycin resistance gene (*hygR*) using primers pForHyg6902 and pRevHyg6902. To construct MGM12 and MGP12, vector pCRISPomyces-2_Δ*sco3390-3389_hygR* was conjugated into the wild type (M145) and TopA-controlled (PS04) *S. coelicolor* strains, respectively. Hygromycin-resistant exconjugants were selected on the R2-S agar (R2 agar without sucrose), and the lack of *sco3390-3389* genes was verified by PCR reaction

using seq_3390_fw and sco3388_seq_rv oligonucleotides (product length 762 bp in deletion strains, and 2696 bp for the wild-type). Next, the deletion was confirmed by chromosome isolation and sequencing of PCR product amplified using seq_3390_fw and delTCS_outside_rv oligonucleotides. Next, pCRISPomyces-2_Δ*sco3390-3389* plasmid was eliminated from the verified obtained strain by cultivation at 39 °C for 2 days, followed by replica plating on selective and nonselective plates to exclude restoration of hygromycin sensitivity.

For SCO3389 overproduction plasmid pIJ10257_*sco3389* was constructed, in which gene *sco3389* encoding response regulator was cloned under control of constitutive p*ermE* promoter. To this end, we used oligonucleotides 3389_NdeI_fw and 3389_XhoI_rv to amplify *sco3389* gene using M145 chromosomal DNA as a template. Next, we cloned PCR product into pIJ10257 (6) using NdeI and XhoI sites yielding pIJ10257_*sco3389* plasmid. To construct MGM11 and MGP11, the wild type (M145) and PS04 *S. coelicolor* strains, respectively, were conjugated with the pIJ10257_*sco3389* plasmid, according to the procedures described by (7) and subsequently selected for hygromycin resistance. Obtained strains were confirmed by PCR reaction using ermEp_fw and 3389_XhoI_rv oligonucleotides.

For construction of MGM14 and MGP14 strains we first performed frame-shift knockout of the *sco3390* gene using CRISPR-Cas9 procedure and pCRISPomyces-2 plasmid according to the procedure described in (4). To this end we used oligo3390_BbsI_fw and oligo3390_BbsI_rv oligonucleotides to introduce double strand break into *sco3390* gene. The oligonucleotides were cloned into pCRISPomyces-2 vector yielding pCRISPomyces-2_oligo3390 using Golden Gate assembly (4). Next, we designed homology recombination template deleting 19 nucleotides at the beginning of *sco3390* gene and inducing a frame-shift. The template was amplified using

oligonucleotides Del3390_fr1_XbaI_fw, Del3390_fr1_rv, Del3390_fr2_fw, Del3390_fr2_XbaI_rv. The obtained fragments (length 1020 bp and 1040 bp, respectively) were then used as template for overlapping PCR, using external Del3390_fr1_XbaI_fw and Del3390_fr2_XbaI_rv oligonucleotides. PCR product was cloned into pCRISPomyces-2_oligo3390 using XbaI site, yielding pCRISPomyces-2_Δ*sco3390* plasmid. PCR targeting method (5) was used to exchange apramycin resistance gene in pCRISPomyces-2_Δ*sco3390* plasmid for hygromycin resistance gene (*hygR*). Vector pCRISPomyces-2_Δ*sco3390_hygR* was then conjugated into WT (M145) and TopA-controlled (PS04) *S. coelicolor* strains, yielding MGM10 and MGP10, respectively. Hygromycin-resistant exconjugants were selected on the R2-S agar (R2 agar without sucrose), and the short deletion within *sco3390* gene was verified by chromosome isolation and sequencing of PCR product amplified using seq_3390_fw oligonucleotide. Next, pCRISPomyces-2_ Δ*sco3390* plasmid was eliminated from the obtained strains by cultivation at 39 °C for 2 days, followed by replica plating on selective and nonselective plates to exclude restoration of hygromycin sensitivity. Next, pIJ10257_*sco3389* plasmid was conjugated to the obtained strains, yielding MGM14 and MGP14.

To construct MB01 and MB02 strain the deletion of *sco4667* and *sco4668* was performed using PCR-targeting procedure described earlier (5). To this end, hygromycin resistance cassette was amplified using primers Del4667_PacI_FW and Del4668_PacI_RV, and vector pIJ10700 as a template. Modified cosmid StD40A was then introduced by conjugation into M145 wild type and PS04 strain (selection on hygromycin), yielding MB01 and MB02 strains, respectively. Next, we searched for double cross-over mutants (hygromycin resistant and kanamycin sensitive colonies)

and selected colonies were verified by PCR (Flank_up4667_FW and Flank_down4668_RV (product length 1730 bp).

In MGP20 strain, transposon mimicking cassette containing hygromycin resistance gene and *oriT* (1583 nt) was inserted into *sco4699* gene directly between 312 and 313 nucleotide of its coding sequence. To this end, the cassette was amplified using tn_mimick_4699_fw and tn_mimick_4699_rv oligonucleotides and vector pIJ10700 as a template. Then, cosmid StD31 was modified by PCR-targeting (5) and introduced into PS04 strain by conjugation (selection on hygromycin MS agar). Next, we searched for double cross-over mutants (hygromycin resistant and kanamycin sensitive colonies) and verified by PCR (hygr_inside and sco4699_start_rv (product length 555 bp), as well as sequencing.

C. Oligonucleotides used in this study

Name	Sequence	Description
Del4667_PacI_FW	GATGGCCGCGCCGCCCGTGGCGGTCCTACGGTGAGCCGCTTAATTAAATTCCGGGGATCCGTCGACC	deletion of *sco4667-4668*
Del4668_PacI_RV	ATCCGTACACCGTCGTTCGCCGCGCGACGCAGAACCGCGTTAATTAATGTAGGCTGGAGCTGCTTC	
Flank_up4667_FW	GGCGCAGTGCGGCAGGGGCTAGAG	verification of *sco4667-4668* deletion

Flank_down4668_RV	GAAGTGCCGCCCCGTGCAGGATTCGTG	
tn_mimic_4699_fw	GGCCGCGTGCTGACGGAGACGGACGCCTTGGGCCGTATTCCGGGGATCCGTCGACC	amplification of transposon mimicking cassette for insertion in *sco4699* gene
tn_mimic_4699_rv	GGTTGTCCTGAGCGTCGTACGTGTACCGGGTTGTATGTAGGCTGGAGCTGCTTC	
Oligo3390_BbsI_fw	ACGCGGCCCAGAACGAGTCGACAC	sgRNA for CRISPR-Cas9 deletion of *sco3389*-*3390* genes
Oligo3390_BbsI_rv	AAACGTGTCGACTCGTTCTGGGCC	
Del3390_fr1_XbaI_fw	GCTCTAGAGTACTCCGGAGTGCACCTCGC	Amplification of template for frame shift mutation of *sco3390*
Del3390_fr1_rv	CTCCGAGCACGACGAAGGCCCCGGGTGTCGGCGGAGGAAGTC	
Del3390_fr2_fw	GACTTCCTCCGCCGACACCCGGGGCCTTCGTCGTGCTCGGAGT	
Del3390_fr2_XbaI_rv	GCTCTAGACCTCCTCGTACAGCTCGTGCG	
seq_oligo3390	GTGTGAAACTTCTGTGAATG	sequencing of proper sgRNA cloning into p-CRISPomyces-2

pForHyg6902	GTGCCGTTGATCGTGCTATG	amplification of hygromycin cassette for exchanging apramycin resistance in pCRISomyces-2_ΔTCS by PCR targeting
pRevHyg6902	CCTTGCCCCTCCAACGTCATCTCGTTCTCCGCTCATGAGCTCAGGCGCCGGGGGCGG	
del_tcs_frI_fw	GCTCTAGACTGGAGCAGCCACCGGAAATC	amplification of recombination template for *sco3390-sco3389* deletion
del_tcs_frI_rv	CACAGTCCCGTCAGCCCTGTCGTAACCCAGCGTAGGGAAGCG	
del_tcs_frII_fw	CGCTTCCCTACGCTGGGTTACGACAGGGCTGACGGGACTGTG	
del_tcs_frII_rv	GCTCTAGAGCAGCTCACGATGCGGATGAC	
seq3390_fw	CACTCGTTGGCGAAGAGCAG	verification of deletion of *sco3389-3390* by PCR on the *S. coelicolor* chromosome
sco3388_seq_rv	GAGGAGACTGAAGTGGTGG	
del_TCS_outside_rv	GCACCTGATGGTCCTGCAC	
sekwmatrpCRISPomyces	GTGCTTTTTACTCCATCTGG	sequencing of editing template after SLIC in pCRISPomyces-2_ΔTCS
sekwmatrpCRISPomyces RV	GAGCGTCGATTTTTGTGATG	
3389_NdeI_fw	GGCATATGGCGATCCGCGTGATGC	

3389_XhoI_rv	GGCTCGAGTCAGCCCTGTCCGCCG CC	amplification of *sco3389* gene for cloning into pIJ10257 vector in NdeI and XhoI sites
rpfAF	GAGTCCGGCGGCAACTGGTC	verification of DNaseI digestion in samples for RNA-seq experiments
rpfAR	GCTGGGACTTGCTCGCCTGG	
hygr_inside	CACGGGACCAACATCTTCG	PCR verification of cloning transposon mimicking casette in MGP20 strain
sco4699_start_rv	CAGATTGCCTGCTGTGTCG	
hrdB_fw	TGCTCTTCCTGGACCTCATC	RT-qPCR experiments
hrdB_rv	GTAGCCCTTGGTGTAGTCGAA	
sco4667RTfw	ACGGGCCCGGTTGGT	
sco4667RTrv	AGGCCGAGGGCCATCGT	
sco4699RTfw	CGCATCGTGGCCGAAT	
sco4699RTrv	GGAGTGTGCCGGTGAATACG	
3543_RTfwd	CCGTTGCGGGCTTTTCCCG	
3543_RTrev	GATCGAAACACAGAACGACC	
3389_RT_fw	CCGGGTTCCGGATGGT	
3389_RT_rv	CATGCGGACGTCCATCAG	
3390_RT_fw	ACCCCCCGGCAACTG	

3390_RT_rv	TGCTTGCGCGTGTTGGT	

References:

1. Bentley,S.D., Chater,K.F., Cerdeño-Tárraga,A.-M., Challis,G.L., Thomson,N.R., James,K.D., Harris,D.E., Quail,M.A., Kieser,H., Harper,D., *et al.* (2002) Complete genome sequence of the model actinomycete Streptomyces coelicolor A3(2). *Nature*, **417**, 141–147.

2. Szafran,M., Skut,P., Ditkowski,B., Ginda,K., Chandra,G., Zakrzewska-Czerwińska,J. and Jakimowicz,D. (2013) Topoisomerase I (TopA) is recruited to ParB complexes and is required for proper chromosome organization during Streptomyces coelicolor sporulation. *J. Bacteriol.*, **195**, 4445–4455.

3. Szafran,M.J., Gongerowska,M., Gutkowski,P., Zakrzewska-Czerwińska,J. and Jakimowicz,D. (2016) The Coordinated Positive Regulation of Topoisomerase Genes Maintains Topological Homeostasis in Streptomyces coelicolor. *J Bacteriol*, **198**, 3016–3028.

4. Cobb,R.E., Wang,Y. and Zhao,H. (2015) High-Efficiency Multiplex Genome Editing of Streptomyces Species Using an Engineered CRISPR/Cas System. *ACS Synth Biol*, **4**, 723–728.

5. Gust,B., Challis,G.L., Fowler,K., Kieser,T. and Chater,K.F. (2003) PCR-targeted Streptomyces gene replacement identifies a protein domain needed for biosynthesis of the sesquiterpene soil odor geosmin. *Proc Natl Acad Sci U S A*, **100**, 1541–1546.

6. Hong,H.-J., Hutchings,M.I., Hill,L.M. and Buttner,M.J. (2005) The role of the novel Fem protein VanK in vancomycin resistance in Streptomyces coelicolor. *J Biol Chem*, **280**, 13055–13061.

7. Kieser,T., Bibb,M.J., Buttner,M.J., Chater,K.F. and Hopwood,D.A. (2000) Practical Streptomyces genetics The John Innes Foundation, Norwich.
